# Promotion of arsenic phytoextraction efficiency in the fern *Pteris vittata* by the inoculation of As-resistant bacteria: a soil bioremediation perspective

**DOI:** 10.3389/fpls.2015.00080

**Published:** 2015-02-18

**Authors:** Silvia Lampis, Chiara Santi, Adriana Ciurli, Marco Andreolli, Giovanni Vallini

**Affiliations:** ^1^Department of Biotechnology, University of VeronaVerona, Italy; ^2^Department of Agricultural, Food and Agro-Environmental Sciences, University of PisaPisa, Italy

**Keywords:** arsenic, arsenopyrite cinders, phytoextraction, plant growth-promoting rhizobacteria, *Pteris vittata*, rhizosphere-enhanced phytoremediation

## Abstract

A greenhouse pot experiment was carried out to evaluate the efficiency of arsenic phytoextraction by the fern *Pteris vittata* growing in arsenic-contaminated soil, with or without the addition of selected rhizobacteria isolated from the polluted site. The bacterial strains were selected for arsenic resistance, the ability to reduce arsenate to arsenite, and the ability to promote plant growth. *P. vittata* plants were cultivated for 4 months in a contaminated substrate consisting of arsenopyrite cinders and mature compost. Four different experimental conditions were tested: (i) non-inoculated plants; (ii) plants inoculated with the siderophore-producing and arsenate-reducing bacteria *Pseudomonas* sp. P1III2 and *Delftia* sp. P2III5 (A); (iii) plants inoculated with the siderophore and indoleacetic acid-producing bacteria *Bacillus* sp. MPV12, *Variovorax* sp. P4III4, and *Pseudoxanthomonas* sp. P4V6 (B), and (iv) plants inoculated with all five bacterial strains (AB). The presence of growth-promoting rhizobacteria increased plant biomass by up to 45% and increased As removal efficiency from 13% without bacteria to 35% in the presence of the mixed inoculum. Molecular analysis confirmed the persistence of the introduced bacterial strains in the soil and resulted in a significant impact on the structure of the bacterial community.

## INTRODUCTION

Arsenic is widely dispersed in the Earth’s crust with an average concentration of ∼5 mg kg^-1^. It is a component of more than 200 minerals, although it primarily exists as arsenopyrite and other sulfides. Rocks can release arsenic compounds during weathering, allowing dispersion by wind and water. The natural arsenic content of soils ranges from 0.01 to more than 600 mg kg^-1^ (Yan-Chu, 1994). Approximately one third of the arsenic in the atmosphere is also from natural sources, such as volcanoes and forest wildfires (United States Environmental Protection Agency [US-EPA], 1998).

The remaining arsenic in the environment is anthropogenic in origin. Arsenic is used in the pharmaceutical, glass, timber, and leather industries, and for the production of pigments, metal alloys, semiconductors, and optoelectronics. Uncontaminated soils usually contain 0.2–40 mg kg^-1^ arsenic but concentrations of 100–2500 mg kg^-1^ can be found in the vicinities of copper-smelting plants and in heavily pesticide-contaminated agricultural soils, which are the greatest sources of arsenic pollution (World Health Organization [WHO], 2000). The diverse industrial uses of arsenic provide many opportunities for human exposure to the element (Garelick et al., 2008). Arsenic in soils exists predominantly as arsenate (AsV), which includes HAsO_4_^2-^ and H_2_AsO_4_^-^. However, arsenite (AsIII), arsine (AsH_3_), and several organoarsenic compounds are also found (Roy et al., 2015).

Arsenic is acutely toxic to humans and also has a chronic impact on health, as well as genotoxic and carcinogenic effects (Léonard and Lauwerys, 1980; Ratnaike, 2003; Hughes et al., 2011). It is considered to be five times as dangerous as lead (United States Department of Health and Human Services [US-DHHS], 2007). The chronic effects of arsenic include gastrointestinal disorders, anemia, peripheral neuropathy, skin lesions, hyperpigmentation, gangrene of the extremities, vascular lesions, liver and kidney damage, and spontaneous abortions (Szymañska-Chabowska et al., 2002; Fernández et al., 2012). The inhalation of arsenic-containing compounds is a minor exposure route with the exception of workers in the copper-smelting and pesticide-manufacturing industries, and in power plants burning arsenic-rich coal (Naujokas et al., 2013). Arsenic exposure through contaminated drinking water is common in mine drainage areas and where the bedrock has a high arsenic content (Nordstrom, 2002; Rahman et al., 2009) exceeding the 10 μg l^-1^ safety limit established by the United States Environmental Protection Agency (US-EPA, 1998) and the World Health Organization (WHO, 2000). Arsenic may also be present in the diet, particularly in seafood, e.g., marine fish, mussels, and certain crustaceans (European Food Safety Authority [EFSA], 2009).

The presence of arsenic in the environment and its associated health risks have led to the deployment of conventional remediation strategies for the cleanup of contaminated sites including removal (excavation and landfilling) and containment (capping). Because both these approaches are expensive, plant-assisted bioremediation (phytoremediation) has been considered as an inexpensive and environmentally beneficial *in situ* treatment for polluted soils (Pilon-Smits, 2005). This is based on the ability of hyperaccumulator plants to extract metals (including metalloids such as arsenic) from contaminated soils and sequester the minerals in their aboveground biomass (Lasat, 2002). However, effective phytoremediation in metal/metalloid-contaminated soils requires a detailed understanding of the complex interactions in the rhizosphere, because soil microbes influence metal bioavailability (Rani and Juwarkar, 2013). For example, microbes catalyze redox reactions leading to changes in the mobility of metals and their ions, and thus the efficiency with which they are taken up by roots (Sessitsch et al., 2013). Microbes therefore play a crucial role in arsenic geochemical cycling through biochemical transformation, e.g. reduction, oxidation, and methylation (Smedley and Kinniburgh, 2002; Lloyd and Oremland, 2006; Páez-Espino et al., 2009).

Here we focus on a severe case of arsenic contamination in the Scarlino industrial area (south–west Tuscany, GR, Italy) caused by the dumping of 1.5 million tons of arsenopyrite cinders generated during the manufacture of sulfuric acid. The cinder layer covering the soil is currently being removed as the first step toward restoring the site, but a more refined strategy is required to regenerate the underlying soil, which is now heavily contaminated with arsenic minerals. We tested a remediation strategy for soil mixed with arsenopyrite cinders based on microbially enhanced phytoextraction using the arsenic hyperaccumulator fern species *Pteris vittata*. We carried out a mesocosm experiment under glasshouse conditions as a preliminary test to evaluate the efficiency of arsenic phytoextraction by *P. vittata* with or without the help of bacterial inoculums comprising species isolated from the rhizosphere of autochthonous plants grown on surrounding soil. The bacteria were enriched by selection with arsenite As(III) or arsenate As(V) to identify species that are arsenic resistant, able to reduce arsenate to arsenite, and able to promote plant growth by producing indoleacetic acid (IAA) or siderophores. The overall aim was to identify bacterial strains that promote the translocation of arsenic from contaminated environmental matrices into plant tissues, especially the epigeal portion of *P. vittata*.

## MATERIALS AND METHODS

### THE CONTAMINATED SITE

**FIGURE 1:**
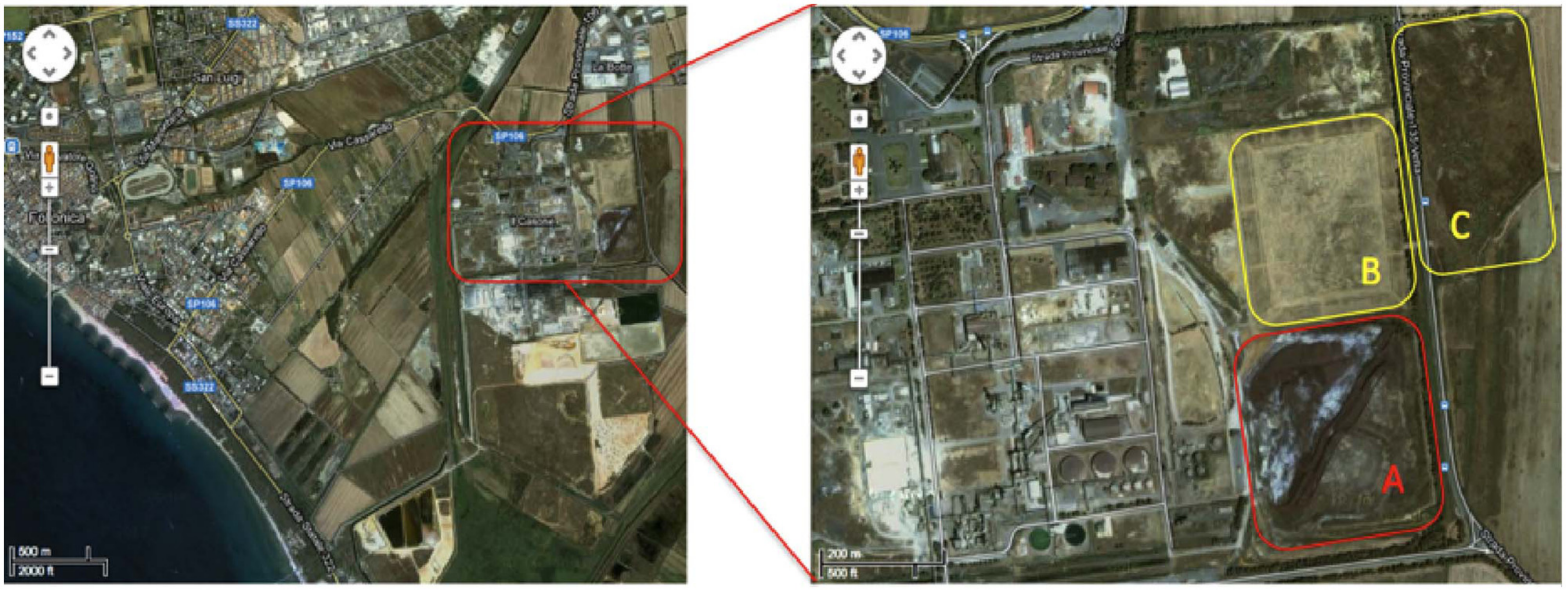
**Satellite map of the Scarlino industrial area.** Letters indicate the main contaminated sectors within the site. **(A)** Current 700,000-ton dump of arsenopyrite cinders. **(B)** Disposal field for fine arsenopyrite particles. **(C)** Former lagoon for pyrite enrichment sludge.

The contaminated site is located in the Scarlino industrial area (Province of Grosseto, south–west Tuscany, GR, Italy) adjacent to a former Nuova Solmine SpA sulfuric acid production facility that was operational between 1962 and 1995. The production method involved the roasting of arsenopyrite mined from the Colline Metallifere source, 20 km to the east of the processing plant (**Figure 1**). The Nuova Solmine SpA site has been classified by the Regional Government of Tuscany as an industrial landfill suitable for reclamation (Site GR66, Resolution No. 384, 21 December, 1999; Ciurli et al., 2014). During the operational lifetime of the facility, ∼1.5 million tons of arsenopyrite cinders with an average arsenic content of 370 mg kg^-1^ was dumped in the landfill site, exposing ∼550 tons of arsenic to rainfall and creating a serious risk of leaching and groundwater contamination (Focardi and Tiezzi, 2009). Nuova Solmine SpA is facilitating the reclamation of this site by excavating the cinders and reusing them for industrial processes such as steel production and brick manufacturing, leaving 700,000 tons of cinders remaining on site. These remnants have sunk 2–5 m below the ground level, so the excavated field is being progressively refilled with clean agricultural cover soil from a nearby site to prevent the dispersion of contaminated dust.

### ISOLATION OF ARSENIC-RESISTANT BACTERIAL STRAINS FROM ENRICHMENT CULTURES

Soil aliquots (5 g) from samples collected within the rhizosphere of different autochthonous plants growing in the Scarlino area were incubated in 250-ml Erlenmeyer flasks containing 100 ml R2A liquid medium (Reasoner and Geldreich, 1985) in the presence of 2 mM As(III) or As(V). All enrichment cultures were prepared in duplicate and maintained for 2 weeks at 27°C in the dark on an orbital shaker at 200 rpm. For the isolation of arsenic-resistant bacteria, appropriate dilutions of each enrichment culture were plated on R2A agar medium and incubated at 27°C for 5 days. Colonies with different morphotypes were picked from the plates at the end of the incubation period and repeatedly streaked until axenic cultures were obtained. Pure cultures of each isolate were stored in 30% (w/v) glycerol at –80°C.

### DETERMINATION OF MINIMUM INHIBITORY CONCENTRATIONS (MICs) FOR ARSENITE AND ARSENATE

The bacterial isolates were streaked from liquid culture aliquots onto R2A agar medium in Petri dishes supplemented with increasing concentrations of arsenite (1–50 mM) and arsenate (10–100 mM). The plates were then incubated at 27°C for 5 days. The growth of the different colonies was verified by referring to reference plates lacking arsenic compounds.

### ANALYSIS OF PLANT GROWTH-PROMOTING TRAITS

#### Assay for 1-amino-cyclopropane-1-carboxylic acid (ACC) deaminase activity

Bacterial isolates with high MIC values for both arsenite and arsenate were assayed for their ability to promote plant growth. Each strain was grown for 48 h in 4 ml DF minimal medium (Penrose and Glick, 2003) containing 2 g l^-1^ (NH_4_)_2_SO_4_ as a nitrogen source. The cells were then collected by centrifugation (5000 rpm, 5 min, 4°C), washed twice with 0.9% NaCl and inoculated into 30 ml DF minimal medium without a nitrogen source to achieve an optical density at 600 nm (OD) of 0.1. After 2 days, 1 ml of the culture was transferred to a second flask containing 30 ml DF minimal medium and this step was repeated until no further growth was detected in the absence of a nitrogen source. The cells were harvested by centrifugation as above and divided into three flasks containing 30 ml DF minimal medium, DF medium containing 2 g l^-1^ (NH_4_)_2_SO_4_ or DF medium supplemented with 3 mM ACC. The latter is heat labile and was therefore prepared as a 0.5 M stock, sterilized by passing through a 0.2-μm filter membrane (Millipore) and frozen in small aliquots at –20°C which were thawed just before use. The cultures were incubated on an orbital shaker (250 rpm) in the dark and checked for growth after 7 days.

#### Assay for IAA production

The bacterial strains were cultured for 5 days in R2A medium containing 0.5 mg ml^-1^ tryptophan, a precursor of IAA. After 2 and 5 days of incubation, 1 ml of each suspension was mixed vigorously with 2 ml Salkowski’s reagent and incubated at room temperature for 20 min before checking for the appearance of pink coloring, which indicated the presence of IAA (Cavalca et al., 2010). The quantity of IAA produced by 10^7^ CFU ml^-1^ of each suspension was determined as previously reported (Glickmann and Dessaux, 1995).

#### Assay for siderophore production

Siderophore production was detected by streaking bacterial isolates on blue agar plates containing Chromeazurol S (CAS; Sigma–Aldrich) and incubating at 27°C for 5 days before checking for orange halos around the colonies, as described by Schwyn and Neilands (1987).

### *IN VITRO* ARSENATE REDUCTION TEST

The ability of bacterial isolates to reduce As(V) was determined by inoculating vials containing 5 mM As(V) in 30 ml Tris minimal medium (Sokolovská et al., 2002) and incubating at 27°C for 72 h. At each sampling point, 1 ml of the suspension was used to determine cell growth based on OD values, and the As(III) and As(V) concentrations were determined by spectrophotometry according to Cummings et al. (1999). Control vials without bacteria were used to account for potential abiotic arsenate reduction.

### TAXONOMIC ANALYSIS OF BACTERIAL ISOLATES

Bacterial isolates that promoted plant growth and/or showed resistance to high concentrations of both As(III) and As(V) were analyzed by *16S rRNA* gene sequencing. DNA was isolated using the beadbeater method (Lampis et al., 2014), and the *16S rRNA* genes were amplified by PCR using primers F8 and R11 (Weisburg et al., 1991) under the following conditions: initial denaturation at 95°C for 5 min followed by 30 cycles of 95°C for 45 s, 52°C for 45 s, and 72°C for 2 min, with a final extension step at 72°C for 5 min. The products were transferred to the pGEM-T vector (Promega, Italy) and both strands were sequenced (Primm, Italy). Phylogenetic neighbors were identified by using BLAST (Altschul et al., 1997) and megaBLAST (Zhang et al., 2000) to search the database of type strains with valid prokaryotic names. The 50 sequences with the highest scores were then used to calculate pairwise sequence similarity using a global alignment algorithm available on the EzTaxon server (http://www.ezbiocloud.net/eztaxon; Kim et al., 2012). Multiple sequence alignments were carried out using ClustalW v1.83 (Thompson et al., 1997). Phylogenetic trees were constructed using the neighbor-joining method in MEGA v5.0 (Tamura et al., 2011) with 1000 data sets examined by bootstrapping. Missing nucleotides at the sequence termini were not included.

### PHYTOEXTRACTION EXPERIMENTAL DESIGN AND TEST CONDITIONS

#### Botanical species and pot experiments

The arsenic hyperaccumulator *P. vittata* (Chinese brake fern) was initially propagated as prothalli from spores in growth chambers under controlled environmental conditions (25°C, 65–70% relative humidity, 100 μmol m^-2^ s^-1^ photosynthetically active radiation, 16-h photoperiod) to yield the sporophytes used in the pot experiments. The sporophytes were cultivated in pots containing 3 kg soil under glasshouse conditions (25°C, 65–70% relative humidity, 350 μmol m^-2^ s^-1^ photosynthetically active radiation, 16-h photoperiod). *P. vittata* plants were cultivated either in unpolluted soil (agricultural cover soil, C) or in a contaminated soil (the amended matrix, M) consisting of arsenopyrite cinders from the Scarlino site mixed with 30% (w/w) mature compost from the aerobic stabilization of source-separated household organic waste by windrow composting with forced aeration and periodic turning. The soil was sterilized by autoclaving at 121°C for 15 min before use.

Both the C and M soils were tested with four different treatments, each with five replicates: (i) non-inoculated plants; (ii) plants inoculated with the siderophore-producing, arsenate-reducing bacterial strains P1III2 and P2III5 (inoculum A); (iii) plants inoculated with the siderophore and IAA-producing bacterial strains MPV12, P4III4, and P4V6 (inoculum B); and (iv) plants inoculated with A + B (inoculum AB). The experiment lasted 4 months and the inoculums were applied at a final concentration of 10^8^ CFU g^-1^ soil, either at the beginning of the experiment or after 2 months. Before inoculation, the total bacterial count of the M and C soils was determined by plating aliquots of soil aqueous suspensions onto R2A agar and incubating at 27°C in the dark for 5 days. Bacterial growth was measured by counting the colony forming units (CFUs). At the end of the experiment, the plants were harvested and dissected into hypogeal (root) and epigeal (frond) portions. Soil samples were collected from each pot at the beginning (*T* = 0) and end (*I* = 1) of the test and stored at –80°C.

#### Determination of the arsenic content in the soil and plant tissues

Soil samples were dried at 80°C for 3 days, ground and homogenized, and 1 g of each sample was digested according to a modification of USEPA Method 3051 in an ETHOS 900 microwave system (Milestone, Bergamo, Italy) with pulsed mode emission, as described by Ciurli et al. (2014).

Plant roots and fronds were rinsed with deionized water in an ultrasonic cleaner to remove soil particles, dried at 50°C in an oven for 1 week, weighed and then ground to powder and sieved through a 1-mm mesh screen using a Thomas Wiley Mini-Mill (Thomas Scientific, Swedesboro, NJ, USA). We then digested 0.1–0.5 g of each dried plant sample following the same procedure used for soil.

The arsenic content of each sample was determined by inductively coupled plasma optical emission spectrometry (ICP-OES) using an ELAN 6000 instrument (Perkin-Elmer Corporation, USA). A standard calibration curve was run with each set of samples. Each sample was measured in triplicate and quality control samples were analyzed at the beginning of each test run, after every 10 samples and at the end of each run. Acceptance criteria for the measured concentration was ±5% of the actual concentration. Calibration curves showed excellent linearity, with *r*^2^ values of 0.999 or higher. The arsenic content of the fronds and roots was calculated multiplying the arsenic concentration in each tissue by the corresponding biomass.

#### Bioconcentration factor, translocation factor, and phytoremediation efficiency

The dynamics of arsenic phytoextraction were investigated by measuring the bioconcentration factor (BCF), i.e., the ratio of arsenic concentrations in plant tissues and soil (Brooks, 1998) and the translocation factor (TF), i.e., the ratio of arsenic concentrations in the epigeous and hypogeal portions (Zhang et al., 2002). We also determined the phytoremediation efficiency (PE) using the equation PE = 1–(final arsenic content of soil/initial arsenic content of soil).

#### Statistical analysis

Data were processed by one way analysis of variance (ANOVA) and the difference between specific pairs of mean values was evaluated using Tukey’s test (*P* < 0.05).

### MICROBIAL CHARACTERIZATION OF SOIL SAMPLES BY PCR-DGGE

Total DNA was extracted from soil samples using the FastDNA SPIN Kit for Soil (MP Biomedicals, Santa Ana, CA, USA) according to the manufacturer’s instructions. Approximately 0.5 g of material was used per extraction and the extracted total DNA was then amplified by polymerase chain reaction (PCR) and analyzed by denaturing gradient gel electrophoresis (DGGE), focusing on the V3 hypervariable region of the *16S rRNA* gene (Lampis et al., 2009). The V3 region was amplified using the p2/p3 primer pair (Muyzer et al., 1993) under the following conditions: initial denaturation at 94°C for 5 min followed by 25 cycles of denaturation at 94°C for 30 s, annealing at 57°C for 30 s, and extension at 72°C for 35 s, then a final extension step at 72 °C for 5 min. An 8% gel (19:1 acrylamide/bisacrylamide) was cast using a denaturing gradient of 30–60%, with 100% denaturant defined as 7 M urea and 20% (v/v) formamide. The similarity index was calculated from the DGGE gels using SPSS v8.0 to determine the Pearson coefficient, and NTSYS software was used to construct a dendrogram based on the UPGMA method (Kropf et al., 2004). DGGE bands were excised and incubated for 4 h in 100 μl sterile water before amplification under the conditions described above, except for the use of non-GC-clamped primers. The PCR products were transferred to the pGEM-T vector and sequenced on both strands as above. The sequences were used as BLASTN search queries (Altschul et al., 1997).

## RESULTS

### ISOLATION AND SELECTION OF BACTERIAL STRAINS

More than 80 bacterial strains were isolated as axenic cultures derived from enriched soil samples incubated in the presence of As(III) and As(V). The MIC values were determined by titration and we found that all 80 strains were highly resistant to As(V) but only a few were also resistant to As(III) at concentrations exceeding 5 mM.

The most resistant strains to both As(III) and As(V) were characterized for their ability to promote plant growth. None of the isolates showed significant ACC deaminase activity but five were shown to produce siderophores, namely MPV12, P1III2, P2III5, P4III4, and P4V6 (**Table 1**). Among these five isolates, MPV12 and P4V6 also synthesized IAA, and P1III2, P2III5, and MPV12 were able to reduce arsenate to arsenite in liquid medium. Strain P2III5 was the most efficient, completely reducing 5 mM As(V) in 48 h. Strain P1III2 achieved the complete reduction of 5 mM As(V) in 72 h under both aerobic and microaerophilic conditions. Complete reduction of As(V) occurred when the bacterial strains reached their maximum cell density. There was no evidence of As(V) reduction in the control experiments lacking bacteria, confirming it was solely a microbial process. These five strains were therefore chosen for the phytoremediation experiments.

**Table 1:** List of bacterial strains isolated from arsenic-contaminated matrices in the Scarlino landfill site for roasted arsenopyrite (Tuscany, GR, Italy).

Strain	Closest match in Ez-Taxon database	Identity	MIC (mM)		Plant growth-promoting (PGP) traits		Reduction of As (V)	Inoculum
			As(V)	As(III)	IAA	Siderophores		
P1III2	*Pseudomonas putida* AP013070 *Pseudomonas asplenii* Z76655	99.83% 99.50%	>100	25	–	+	100% reduction of 5 mM As(V) to As(III) in 48 h	A
P2III5	*Delftia lacustris* EU888308	98.73%	>100	10	–	+	100% reduction of 5 mM As(V) to As(III) in 72 h	A
P4III4	*Variovorax boronicumulans* AB300593	99.50%	>100	5	+	+	–	B
P4V6	*Pseudoxanthomonas mexicana* AF273082	100%	>100	5	17.38 ± 1.86 μg ml^-1^	+	–	B
MPV12	*Bacillus thuringiensis ACNF01000156* *Bacillus toyonensis CP006863*	100%	>100	11	12.76 ± 2.12 μg ml^-1^	+	60% reduction of 5 mM As(V) to As(III) in 48 h	B

*The bacteria were selected for phytoextraction tests based on their arsenite/arsenate resistance, As(V) reduction capability, and PGP characteristics.*

### PHYLOGENETIC ANALYSIS OF THE BACTERIAL STRAINS

**FIGURE 2:**
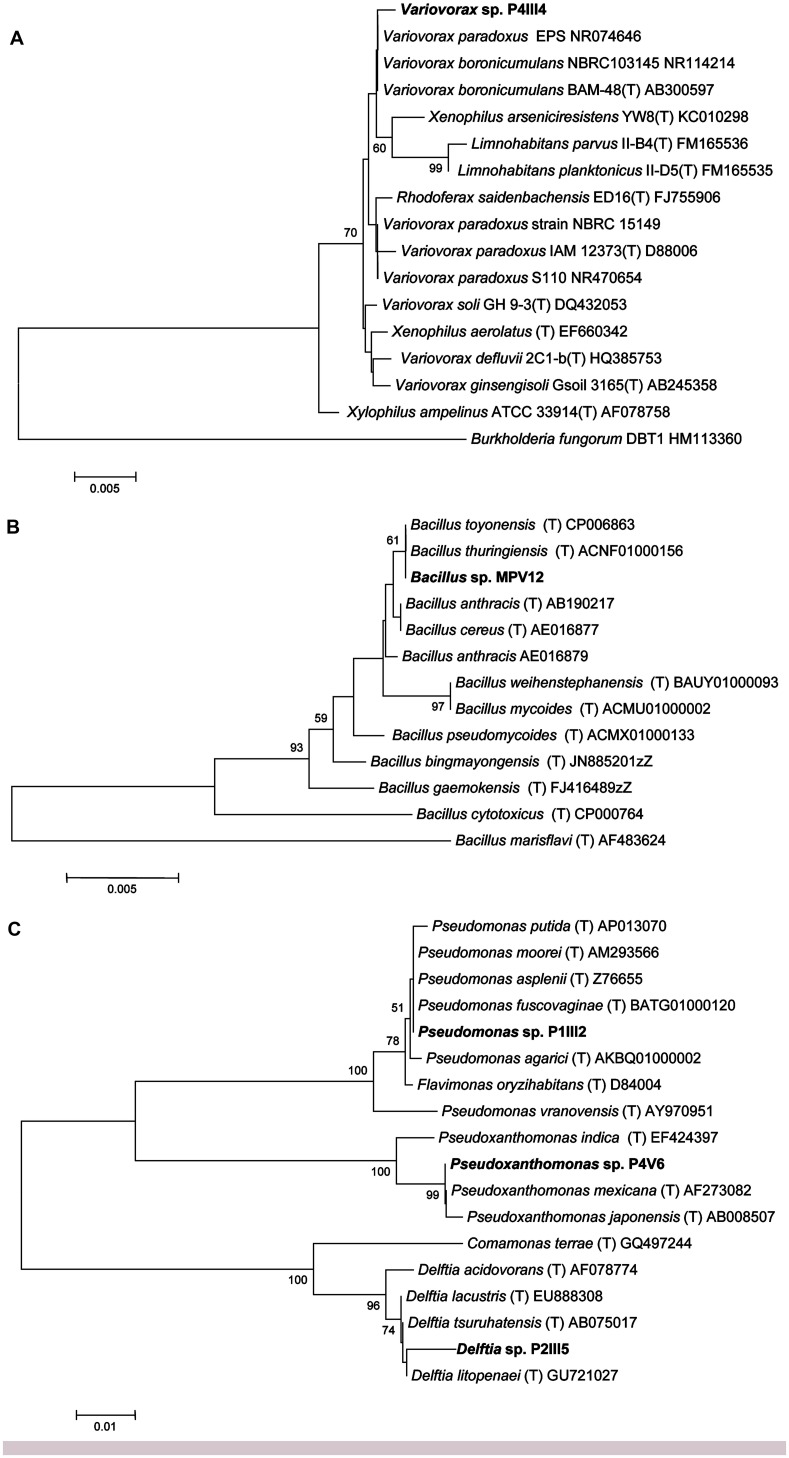
**Neighbor-joining tree inferred using MEGA v5.0 based on the sequences of *16S rRNA* genes, showing the phylogenetic relationships among strains P4IIIA **(A)**, MPV12 **(B)**, P1III2, P4V6, P2III5 **(C)**, and related species.** Bootstrap analysis values for 1000 replicates are shown at the nodes, only for values greater than 50. The scale bars indicate the number of substitutions per nucleotide position.

Sequencing the *16S rRNA* genes from the five selected strains revealed >98.5% identity to reference strains in the Ez-Taxon database (Kim et al., 2012) representing the genera *Bacillus*, *Delftia*, *Pseudomonas*, *Pseudoxanthomonas*, and *Variovorax* (**Figure 2**).

Strain P4III4 showed 99.5% identity to *Variovorax boronicumulans* strain BAM-48(T), a boron-accumulating betaproteobacterium isolated from an experimental field at the University of Tokyo, Japan (Miwa et al., 2008), and 98.5% identity to both *V. paradoxus* strain S110 and *Xenophilus arseniciresistens* strain YW8 (**Figure 2A**). Several *V. boronicumulans* strains are arsenic resistant (Davolos and Pietrangeli, 2013), and *V. paradoxus* is often found in soils contaminated with toxic minerals such as arsenite (Macur et al., 2004) or organic pollutants such as trichloroethylene and polychlorinated biphenyls. Notably, the Scarlino area has a relatively high background content of boron (ARPAT, 2014). *V. paradoxus* is also known to promote plant growth, e.g., *V. paradoxus* strain 5C-2 has been shown to produce ACC deaminase and enhance growth, yield, root length and/or water use efficiency in *Pisum sativum* and *Brassica juncea* (Belimov et al., 2005, 2009).

Strain MPV12 is closely related to the *Bacillus cereus* group (**Figure 2B**) and showed 100% identity to the recently described *B. toyonensis* strain BCT-7112(T) (Jiménez et al., 2013) and *B. thuringiensis* strain ATCC 10792(T). *B. thuringiensis* is often found in arsenic-polluted soils (Oliveira et al., 2008; Villegas-Torres et al., 2011) and is well known for its ability to promote plant growth (Kumar et al., 2011).

Strain P1III2 showed 99.8% identity to *Pseudomonas putida* and 99.5% identity to *P. asplenii* (**Figure 2C**). *Pseudomonas* sp. are As(III)-resistant gammaproteobacteria (Cai et al., 2009) that are often found in metal-polluted soils (Roosa et al., 2014) and are known to promote plant growth (Glick, 2010). Strain P2III5 showed 98.7% identity to *Delftia lacustris,* a betaproteobacterium (**Figure 2C**). *Delftia* sp. are also found in soils polluted with As(III) (Cai et al., 2009). *D. tsuruhatensis* HR4 isolated from the rhizoplane of rice (*Oryza sativa* L., cv. Yueguang) in North China has been shown to fix nitrogen and suppress the growth of plant pathogens (Han et al., 2005). Strain P4V6 showed 100% identity to *Pseudoxanthomonas mexicana*, a gammaproteobacterium (**Figure 2C**) that promotes plant growth and has been isolated from rice grown in arsenic-polluted soil (Bachate et al., 2009).

### PHYTOREMEDIATION WITH NON-INOCULATED AND INOCULATED *P. vittata* PLANTS

*Pteris vittata* plants were cultivated on control agricultural soil (C) or a mixture of arsenopyrite cinder and compost (M) for 4 months. Four experimental treatments were carried out: (i) non-inoculated plants; (ii) inoculum A comprising *Pseudomonas* sp. P1III2 and *Delftia* sp. P2III5, both producing siderophores and capable of arsenate reduction *in vitro*; (iii) inoculum B comprising *Variovorax* sp. P4III4, *Pseudoxanthomonas* sp. P4V6, and *Bacillus* sp. MPV12 all producing siderophores and IAA; and (iv) inoculum AB, comprising all five bacterial strains. The average bacterial count in the soil before inoculation was 10^7^ CFU per gram dry weight.

**FIGURE 3:**
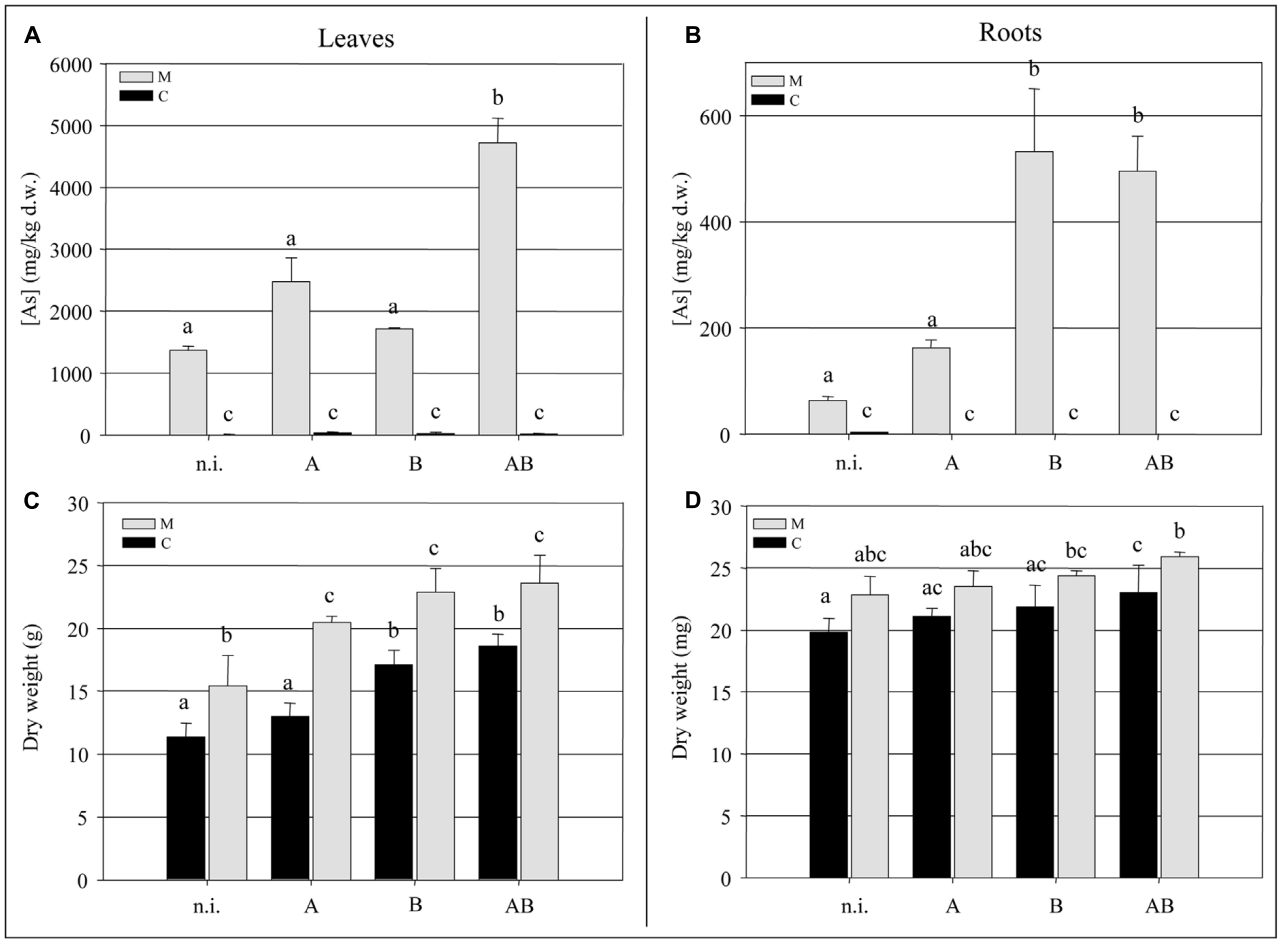
**(A)** Arsenic concentration (mg kg^-1^ dry weight) in *Pteris vittata* fronds. **(B)** Arsenic concentration (mg kg^-1^ dry weight) in *P. vittata* roots. **(C)** *P. vittata* frond biomass (g dry weight). **(D)** *P. vittata* root biomass (mg dry weight). Data are shown for plants grown in either agricultural soil (C) or on arsenic-contaminated soil (M), inoculated (with A, B, or AB) or not inoculated (n.i.).

At the end of the experimental trial, we measured the biomass and arsenic content of the fronds and roots. We observed a significant increase (*P* < 0.05) of ∼35% in the frond biomass of plants grown in the M soil in the presence of bacteria compared to the non-inoculated plants (**Figure 3C**). The bacteria also promoted frond growth in the C soil, with the greatest enhancement of ∼30% achieved by inoculum B and inoculum AB (*P* < 0.05). Interestingly, non-inoculated plants growing in the M soil accumulated significantly more frond biomass (*P* < 0.05) than those growing in the C soil, suggesting that both the soil and the bacteria had an impact on the growth of epigeal tissues. Neither the soil type nor the presence or absence of bacteria appeared to have a significant impact on root biomass (**Figure 3D**).

Inoculum AB promoted a significant increase (*P* < 0.05) in the accumulation of arsenic in the fronds of plants grown in the M soil, resulting in an average concentration of 4700 mg kg^-1^ which represented a 3.5-fold increase over the non-inoculated plants (**Figure 3A**). The arsenic content of the roots was enhanced by inoculum B and inoculum AB, reaching concentrations of up to 500 mg kg^-1^ which represented an eightfold increase over the non-inoculated plants (**Figure 3B**).

**FIGURE 4:**
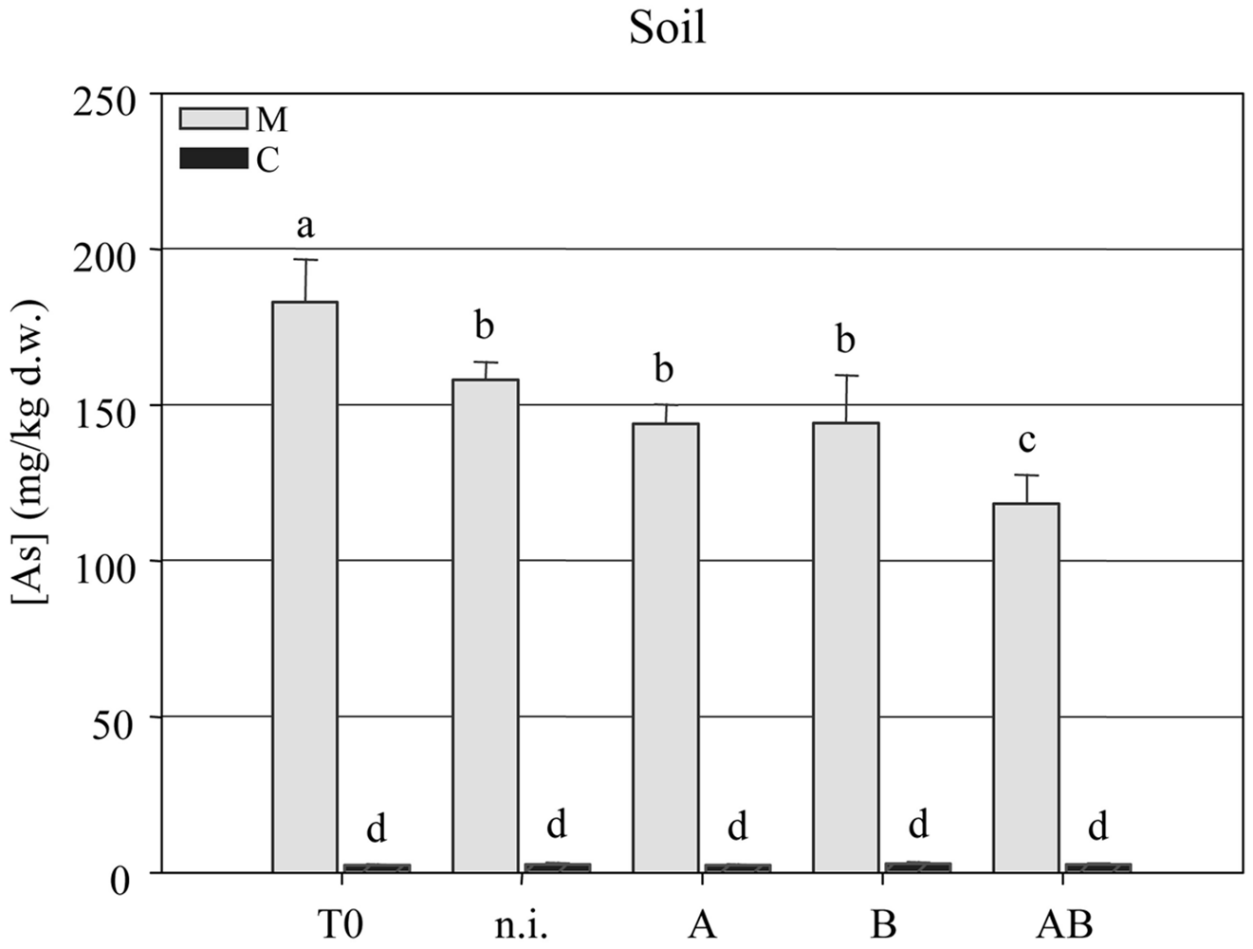
**Arsenic concentration (mg kg^-**1**^ dry weight) in clean agricultural soil (C) and arsenic-contaminated soil (M) at the beginning (*T* = 0) and end (*T* = 1) of the experimental trials with *P. vittata* augmented with inoculums A, B, and AB or not inoculated (n.i.)**.

The arsenic concentration in the soil was measured at the beginning (*T* = 0) and end (*T* = 1) of the experiment, revealing a significant reduction in all four treatment groups but the most efficient As-removal by plants treated with inoculum AB. This reduced the arsenic content of the soil from 182.9 ± 4.35 mg kg^-1^ at *T* = 0 to 118.2 ± 2.96 mg kg^-1^ at *T* = 1 (**Figure 4**).

The combined positive effect of the bacteria on plant biomass and arsenic uptake resulted in a striking difference between inoculated and non-inoculated plants in terms of arsenic sequestration, with inoculum AB achieving the most efficient mobilization. This resulted in the arsenic content of the fronds increasing from 21.1 ± 1.9 mg in non-inoculated plants to 134.17 ± 7.29 mg in plants treated with inoculum AB (**Table 2**). The bioconcentration and TFs were calculated revealing that the bioconcentration of arsenic was enhanced fourfold in plants treated with inoculum AB compared to non-inoculated plants, reaching an average value of 31. In contrast, the translocation of arsenic decreased in inoculated plants, with the most severe decline (∼80%) observed in plants treated with inoculum B.

The plants treated with different combinations of bacteria all achieved a statistically significant (*P* < 0.05) increase in PE, but the best result (35%) was achieved by inoculum AB, a threefold increase in efficiency compared to the non-inoculated plants.

**Table 2:** Effects of different bacterial inoculums (A, B, and AB) on the final arsenic content of *Pteris vittata* fronds and roots, the bioconcentration factor (BCF), the phytoremediation efficiency (PE), and the translocation factor (TF).

	Arsenic content				
	Fronds (mg)	Roots (μg)	BCF	PE	TF
M	21.1 ± 1.9a	1.44 ± 0.16a	7.48 ± 0.97a	13.6 ± 0.9a	21.69 ± 1.56a
MA	50.59 ± 0.72b	3.82 ± 0.35b	13.52 ± 3.4b	21.34 ± 0.34b	15.21 ± 2.56b
MB	39.33 ± 1.88b	12.96 ± 0.41c	9.39 ± 2.86a	21.26 ± 0.89b	3.23 ± 0.87c
MAB	134.17 ± 7.29c	12.79 ± 0.37c	31.08 ± 5.48b	35.37 ± 1.45c	11.48 ± 1.34b

*Within each column, means with the same letter are not significantly different according to Tuckey’s test (*P* < 0.05).*

### ANALYSIS OF THE SOIL BACTERIAL COMMUNITY BY PCR-DGGE

Polymerase chain reaction-denaturing gradient gel electrophoresis analysis was carried out on soil samples collected at the beginning and end of the experimental trials to monitor the persistence of the inoculated strains and to evaluate potential changes in the bacterial community.

**FIGURE 5:**
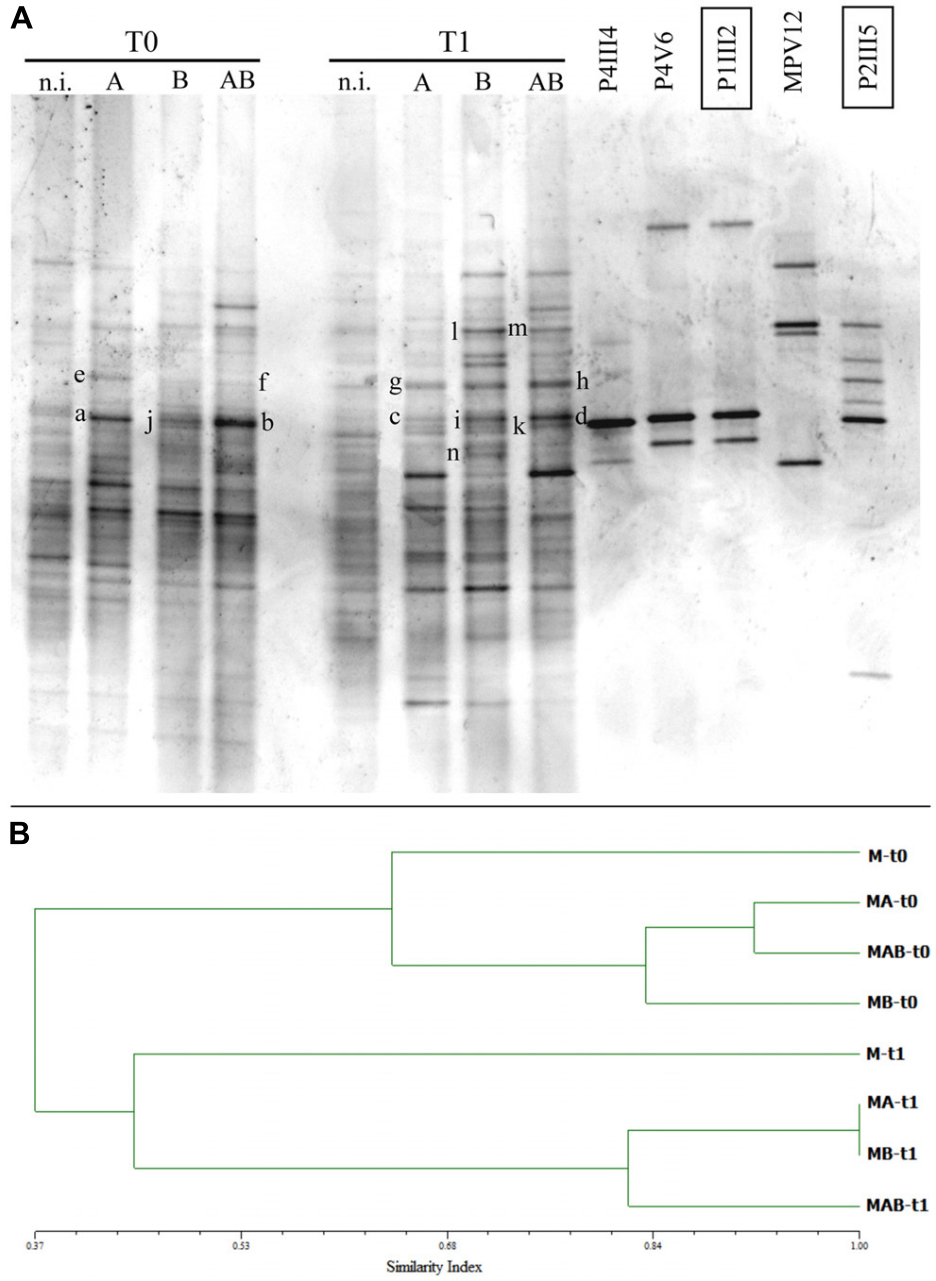
**(A)** Denaturing gradient gel electrophoresis (DGGE) profiles of bacterial communities collected at the beginning (*T* = 0) and end (*T* = 1) of the experimental trials from the rhizosphere of *P. vittata* plants augmented with inoculums A, B, and AB, or not inoculated (n.i.). DGGE profiles of bacterial strains contained in the inoculums are also shown. Letters on the gel indicate bands that were excised and sequenced. **(B)** Dendrogram indicating the similarity indices of the different DGGE profiles.

The DGGE profiles of soil samples collected at each sampling point confirmed that the five inoculated strains persisted in the soil and remained part of the community at the end of the experiment (**Figure 5A**). DGGE bands from the inoculated strains with the same mobility as the reference strains were excised, purified, re-amplified, and sequenced, confirming their identity (data not shown). Specifically, bands *a*, *b*, *c,* and *d* confirmed identity with strain P1III2, bands *e*, *f*, *g,* and *h* with strain P2III5, bands *j*, *k,* and *i* with strain P4III4, bands *l* and *m* with strain MPV12, and band *n* confirmed identity with strain P4V6 (**Figure 5A**).

Similarity indices were calculated using the Pearson correlation coefficient and a dendrogram was constructed based on the UPGMA method (**Figure 5B**) revealing a change in the structure of the bacterial community caused by the presence of the plants and/or the five inoculated bacterial strains. Specifically, the soils inoculated with A, B, and AB each showed a 0.45 similarity index compared to untreated soils, but the changes were not the same with each inoculum. Indeed, the addition of inoculum AB induced bacterial speciation corresponding to a 20% increase in diversity which differed substantially from the effects of inoculums A and B when applied separately.

## DISCUSSION

We have demonstrated that the inoculation of soil with a mixture of bacteria selected for their ability to promote plant growth and the mobility of arsenic compounds can enhance arsenic phytoextraction from highly contaminated environmental matrices by the hyperaccumulator fern species *P. vittata*. Even extreme contamination, such as soil predominantly comprising arsenopyrite cinders from the Scarlino industrial area in Tuscany, can be substantially remediated using this approach. The bacteria were selected for multiple beneficial traits including the production of IAA and siderophores, and the ability to reduce arsenate to arsenite. The inoculation of contaminated soil with five of the best-performing strains achieved an eightfold increase in the arsenic BCF and a threefold increase in PE compared to non-inoculated plants.

The PE increased from 13% in the absence of the selected bacteria to 35% when *P. vittata* plants were augmented with inoculum AB, comprising all five selected bacterial strains. This can be attributed to the ability of the bacteria to withstand particularly adverse experimental conditions. All five strains are indigenous to the contaminated site and have therefore evolved to prosper in an arsenic-rich environment. Our data indicate that the species in inoculums A and B confer overlapping beneficial properties, with inoculum A containing bacteria with the ability to reduce As(V) and inoculum B containing bacteria that produce IAA. The combination of both abilities therefore creates additive benefits to enhance the growth and remediation capacity of the *P. vittata* plants.

The presence of the five selected bacterial strains also had a profound impact on the functional equilibrium of the *P. vittata* rhizobacterial community, as shown by the similarity indices and DGGE molecular fingerprints of soil samples from non-inoculated plants and those treated with inoculums A, B, and AB. The potential of specific bacterial inoculums to promote arsenic accumulation by plants has been described in previous studies. For example, Yang et al. (2012) inoculated *P. vittata* plants with five different allochthonous bacterial strains from the genera *Delftia*, *Comamonas,* and *Streptomyces* that were able to reduce arsenate to arsenite. This resulted in a 50% increase in biomass after 4 months, and an increase in phytoextraction efficiency from 7% without inoculation to 15% with the addition of different bacterial strains. Similarly, we found that inoculation with the arsenate-reducing strains *Pseudomonas* sp. P1III2 and *Delftia* sp. P2III5 (inoculum A) increased the phytoextraction efficiency from 13.6 to 21%. It is well known that rhizosphere microbes influence the mobility of heavy metals in soil by regulating absorption/desorption equilibria, oxidation/reduction reactions, and other mechanisms (Robert and Berthelin, 1986). The bioavailability of heavy metals in soil is known to be influenced by the rhizosphere microbial community, the interaction between microbes and plant roots, and exudates of microbial origin (Tang et al., 2001). Finally, hydroponically grown *P. vittata* has been shown to utilize both arsenite and arsenate, although arsenate is taken up more efficiently because it competes with phosphate (Wang et al., 2002; Tu et al., 2004).

The IAA-producing strains *Variovorax* sp. P4III4, *Pseudoxanthomonas* sp. P4V6, and *Bacillus* sp. MPV12 (inoculum B) elicited a significant increase in both the BCF and the accumulation of arsenic in the fronds, but the TF fell significantly compared to non-inoculated plants and those treated with inoculums A and AB. This probably reflects the ability of the bacteria to accumulate large amounts of intracellular arsenic and thus prevent its uptake into the roots, as previously reported for the arsenic hypertolerant bacterial strain *Bacillus* sp. DJ-1 (isolated from a treatment plant for industrial eﬄuents in India) which can accumulate arsenic at concentrations of up to 9.8 ± 0.5 mg g^-1^ dry weight (Joshi et al., 2009).

All three inoculums we tested also boosted epigeal plant biomass by an average of 45%. Bacteria that promote plant growth do so by synthesizing beneficial compounds or facilitating the uptake of certain nutrients from the soil (Burd et al., 2000; Çakmakçi et al., 2006). They can also prevent or ameliorate plant diseases (Jetiyanon and Kloepper, 2002; Guo et al., 2004). The inoculation of *P. vittata* with arbuscular mycorrhizal fungi can also boost plant growth, e.g., Leung et al. (2013) infected *P. vittata* roots with *Glomus mosseae* and *G. intraradices* strains that are indigenous to soil contaminated with mining waste, not only achieving a higher biomass but also an increase in the TF from 3 to 10. Similar results were obtained by Trotta et al. (2006). Our bacterial inoculums produced IAA, which is a plant growth hormone (Patten and Glick, 2002) and/or siderophores, which facilitate the uptake of nutrients in the presence of competing metals (Burd et al., 2000). A recent study by Jeong et al. (2014) showed that *Pseudomonas aeruginosa* siderophores can effectively form siderophore–arsenic complexes in aqueous solutions. A series of pot experiments was then carried out to investigate the effect of microbial siderophores as iron-chelators on the phytoextraction of arsenic by *P. cretica*. Plants grown in soil supplemented with siderophores accumulated 3.7-fold more arsenic than control plants growing in normal soil (Jeong et al., 2014).

Several experiments have shown that *P. vittata* can phytovolatilize arsenic and this may also contribute to the overall PE because the vapor released from *P. vittata* fronds contains both As(III) and As(V) (Roy et al., 2015). Furthermore, numerous soil bacteria can volatilize arsenic by reducing arsenate and arsenite to arsine and other organoarsenic compounds. For example, a genetically engineered *Pseudomonas putida* strain was shown to volatilize almost completely the initial arsenite component of the soil into organoarsenic compounds (Chen et al., 2014).

We conclude that the inoculation of plants with arsenic-resistant, growth-promoting bacteria that can reduce As(V) to As(III), particularly bacteria that are indigenous to contaminated sites earmarked for remediation, can improve the efficiency of arsenic phytoextraction even by hyperaccumulator plant species such as *P. vittata*. This approach appears to be particularly useful for heavily contaminated sites such as the landfill for arsenopyrite cinders at the Scarlino industrial site considered in this investigation.

## Conflict of Interest Statement

The authors declare that the research was conducted in the absence of any commercial or financial relationships that could be construed as a potential conflict of interest.
